# Impedance-Based Monitoring of Mesenchymal Stromal Cell Three-Dimensional Proliferation Using Aerosol Jet Printed Sensors: A Tissue Engineering Application

**DOI:** 10.3390/ma13102231

**Published:** 2020-05-13

**Authors:** Sarah Tonello, Andrea Bianchetti, Simona Braga, Camillo Almici, Mirella Marini, Giovanna Piovani, Michele Guindani, Kamol Dey, Luciana Sartore, Federica Re, Domenico Russo, Edoardo Cantù, Nicola Francesco Lopomo, Mauro Serpelloni, Emilio Sardini

**Affiliations:** 1 Department of Information Engineering, University of Padova, 35131 Padua, Italy; 2 Laboratory for Stem Cells Manipulation and Cryopreservation, Department of Transfusion Medicine, ASST Spedali Civili, 25123 Brescia, Italy; bianchetti.andrea@gmail.com (A.B.); braga.simona@gmail.com (S.B.); camillo.almici@asst-spedalicivili.it (C.A.); mirella.marini@asst-spedalicivili.it (M.M.); 3 Biology and Genetics Division, Department of Molecular and Translational Medicine, University of Brescia, 25123 Brescia, Italy; giovanna.piovani@unibs.it; 4 Department of Statistics, University of California, Irvine, CA 92697-1250, USA; Mguindan@uci.edu; 5 Department of Mechanical and Industrial Engineering, University of Brescia, 25123 Brescia, Italy; k.dey@unibs.it (K.D.); luciana.sartore@unibs.it (L.S.); 6 Department of Clinical and Experimental Sciences, University of Brescia, Bone Marrow Transplant Unit, ASST Spedali Civili, 25123 Brescia, Italy; f.re002@unibs.it (F.R.); domenico.russo@unibs.it (D.R.); 7 Department of Information Engineering, University of Brescia, 25123 Brescia, Italy; e.cantu@unibs.it (E.C.); nicola.lopomo@unibs.it (N.F.L.); mauro.serpelloni@unibs.it (M.S.); emilio.sardini@unibs.it (E.S.)

**Keywords:** impedance-based cell spectroscopy, aerosol jet printing, 3D monitoring, mesenchymal stromal cells, tissue engineering

## Abstract

One of the main hurdles to improving scaffolds for regenerative medicine is the development of non-invasive methods to monitor cell proliferation within three-dimensional environments. Recently, an electrical impedance-based approach has been identified as promising for three-dimensional proliferation assays. A low-cost impedance-based solution, easily integrable with multi-well plates, is here presented. Sensors were developed using biocompatible carbon-based ink on foldable polyimide substrates by means of a novel aerosol jet printing technique. The setup was tested to monitor the proliferation of human mesenchymal stromal cells into previously validated gelatin-chitosan hybrid hydrogel scaffolds. Reliability of the methodology was assessed comparing variations of the electrical impedance parameters with the outcomes of enzymatic proliferation assay. Results obtained showed a magnitude increase and a phase angle decrease at 4 kHz (maximum of 2.5 kΩ and −9 degrees) and an exponential increase of the modeled resistance and capacitance components due to the cell proliferation (maximum of 1.5 kΩ and 200 nF). A statistically significant relationship with enzymatic assay outcomes could be detected for both phase angle and electric model parameters. Overall, these findings support the potentiality of this non-invasive approach for continuous monitoring of scaffold-based cultures, being also promising in the perspective of optimizing the scaffold-culture system.

## 1. Introduction

Nowadays, three-dimensional (3D) cell cultures represent a widespread approach adopted in several fields, ranging from pharmaceutics to regenerative medicine. The possibility of reproducing the conditions undergone by cells in living tissues plays a fundamental role in improving the effectiveness of drug testing and tissue engineering [1]. From this perspective, regenerative medicine strategies can be focused on the restoration of damaged tissue by using human mesenchymal stromal cells (hMSCs) in combination with 3D scaffolds [2]. hMSCs were reported to promote tissue repair thanks to several key characteristics, such as their ability to migrate towards injured tissues, their immunomodulatory action and their trophic effect [3]. The combination of bioengineered scaffolds with hMSCs represents one of the most attractive strategies, since it combines the structural, biological and chemical properties of the scaffolds with the possibility of modulating hMSC activities [4]. In recent years, among the multitude of biomaterials used to support hMSCs, hydrogels have been back in the limelight due to their inherently tunable characteristics and their ability to mimic native extracellular matrix (ECM). Several types of hydrogel are available, so far. The solutions reported mainly include both synthetic and natural materials, which present highly desirable features that can be tuned and combined to provide a proper encapsulation of cells, sustain their growth and regulate their fate [5]. Natural hydrogels are often preferred due to their inherent biocompatibility, environmental sensitivity, abundance in source and in binding sites, and thence enhanced interaction with cells. In particular, gelatin and chitosan have been extensively used for tissue engineering applications, since they are biocompatible, biodegradable, non-antigenic, non-toxic and present antimicrobial activity [6,7]. Starting from this evidence and aiming to regenerate bone, recent literature has provided examples of optimized combinations of these materials to realize hybrid hydrogel scaffolds, presenting a structure that mimics native ECM constituents and reporting morphological and mechanical properties suitable to support cell growth, osteo-differentiation and mineralization [8,9,10,11].

From the perspective of optimizing the scaffold-culture system, the need for non-invasive methods able to monitor cell proliferation and differentiation within 3D environments still remains one of the main hurdles to improve scaffold design and better understand cell-material interaction [12,13]. Nowadays, traditional methods adopted for those analytical evaluations (e.g., dyes, DNA sequencing, immune-based assays, fluorescence tags) interfere, in most cases, with the cellular cycle. Therefore, most of these approaches make it fairly impossible to perform a continuous monitoring of cell culture health, requiring either the sacrifice of the tested samples at defined time points or the introduction of an unaffordable toxic effect. The possibility of non-invasively obtaining information on cell activities within the scaffolds—strictly avoiding affecting their vitality and proliferation—would allow a step forward to be made in terms of understanding complex pathophysiological processes.

Since the early 1970s, the use of different physical cues (e.g., optical, electrical, magnetic) has been investigated in order to achieve a completely non-invasive approach. Among all the proposed methods, electrochemical sensors, which correlate electrical parameter variations with specific events of a cell cycle, dominated as the most attractive strategy in term of non-invasiveness, accuracy, sensitivity and cost/time effectiveness [14].

Among the available electrical-based cell monitoring techniques, impedance-based solutions represent one of the most adopted approaches, easily integrable with labware and microfluidic devices [15]. Furthermore, this methodology can be applied to a large variety of cells—even non excitable ones—, thus representing a powerful solution with respect to potential-based measurement systems. This approach is based on the hypothesis that cellular membranes act as insulating elements when seeded on conductive electrodes. Thus, by measuring the impedance changes between two electrodes, information related to cell number, shape and their motility can be obtained [16]. Starting from the reliability already demonstrated by this technique in 2D cell culture—which led also to commercially available impedance-based systems [17]—in the last decades a huge interest has been addressed to the possibility of translating this approach towards 3D settings [18].

In shifting this method from a 2D to a 3D system, several new technical challenges must be considered, since the scaffold material acts as an intercalant in between microelectrodes and the sample, thence playing a crucial role in the overall impedance measured. Considering these aspects, two possible approaches can be highlighted from the literature: (1) making the scaffold conductive or (2) monitoring cell and scaffold dielectric properties by introducing external conductive elements in contact with the scaffold itself. The first option represents the most convenient in terms of electronic performances, reducing the overall contribution of the scaffold to system impedance and thus enhancing the overall sensitivity towards cells. However, as reported by Kahn et al. [19], although several biomaterials integrating conductivity with biocompatibility and biodegradability have been proposed, further research is still required to achieve solutions that are able to offer the very same positive effect that non-conductive scaffolds can provide to cell development.

Due to these reasons, the use of external sensors appears an acceptable compromise to integrate a suitable environment for hMSCs with a minimally invasive sensing strategy. By pursuing this approach, we hypothesized that useful information about cell proliferation and adhesion within the scaffold can be obtained without any detrimental effect on the culture itself. To our knowledge, only a few examples have been proposed for the investigation of scaffold-based 3D cell cultures, mainly adopting metal based electrodes [20,21,22]. This technology was thus demonstrated to be fairly promising to monitor stem-cell proliferation and differentiation state when culturing stem-cell progeny, as—for example—in hMSC expansion [23]. Although acceptable sensitivities and customizable solutions were reported [24,25], all the designs proposed were not easily integrable into the widespread multi-well plates commonly used during routine cell culture. Furthermore, currently employed techniques are still based on the classic photolithographic procedure, thus involving long time and high costs of production. A promising path to overcome these issues is represented by printed electronics, an approach which allows the production of completely customizable sensors, with low costs and high resolution [26]. For instance, screen or inkjet printed materials find plenty of applications in modern electronics [27], biosensing [26,28] and chemical sensing [29]. Compared to those techniques, aerosol jet printing (AJP) allows an improvement both in terms of micrometer-resolution than in terms of the range of usable inks and substrates. Therefore, among the most recent technology used from this perspective, aerosol jet printing (AJP) indeed represents one of the most promising [30,31,32,33].

Therefore, the main goal of this study was to design, develop and validate a reliable impedance-based solution, easily integrable with a traditional multi-well culture system and easily scalable in terms of processing cost and time effectiveness. In more detail, AJP sensors specifically designed to adapt to 96-well plates were produced, folded to create 3D parallel electrodes structures and tested to monitor the adhesion and growth of hMSCs seeded into gelatin-chitosan hybrid hydrogel scaffolds. Reliability of the methodology was assessed in terms of impedance-based results in conjunction with enzymatic outputs and proliferation assays.

## 2. Materials and Methods

### 2.1. Setup Design and Production

The proposed measuring setup was designed in order to fit into a standard plastic 96-well non-treated plate, usually adopted in cells suspension culture and bio-assay experiments.

**Figure 1:**
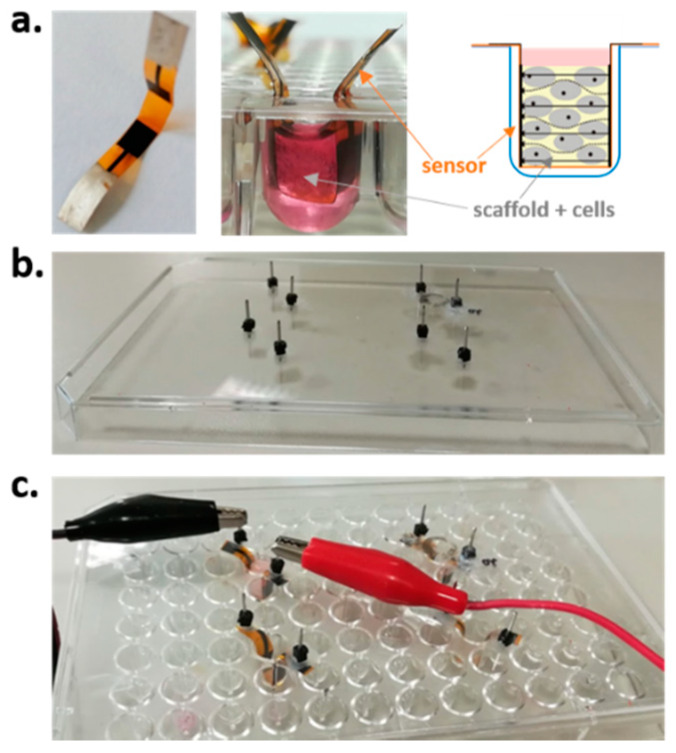
Setup for impedance-based monitoring of human mesenchymal stromal cells (hMSCs) seeded in 3D hybrid hydrogel scaffold: (**a**) Hybrid hydrogel scaffold and aerosol-jet printed (AJP) sensors, (**b**) cap for sterile measurements, (**c**) final measurement setup.

Hybrid gelatin-chitosan hydrogel scaffold were prepared following the procedure described in [10], cut, sterilized using gamma irradiation with Cobalt 60 gamma rays using 27–33 kGy following UNI EN ISO 11137 (Sterilization of Health Care Products) and stored in sealed bags until use. Before sterilization, scaffolds were cut into standard cylinders of 4 mm in diameter and 2 mm in height, in order to ensure the correct fitting within the wells, considering their volumetric expansion when wet. Sensors were realized by means of a commercial AJP system (Aerosol Jet^®^ 300 Series, Optomec, Albuquerque, NM, USA). On the same 20 µm thick polyimide substrate (Kapton^®^ MT from DuPont, Wilmington, DE, USA), two monopolar structures were printed by depositing 3 layers of an electrically conductive carbon-based ink (EXP 2652–28, sheet resistivity 100 Ω/sq./mil) from Creative Materials (Creative Materials Inc., Ayer, MA, USA), in order to achieve a proper electrode conductivity (<1 kΩ). After printing, electrodes were cured in an oven for 30 min at 140 °C. After that, interfacing pads for electrical contacts were realized, in order to limit measurement uncertainty due to possible variability in reproducing the same tips positioning. Square silver pads of 8 × 8 mm were printed on one side of the structure by depositing three layers of silver-based ink (Novacentrix, Austin, TX, USA) and then cured in an oven at 140 °C for 30 min. The final sensors are reported in Figure 1a. Cured sensors were then sterilized in autoclave and stored in sealed bags until use. Sterility during scaffold impedance monitoring was ensured by performing all the measurements under a class A laminar flow hood and using a customized sterile cap. In detail, a cover of a standard 96-well plate was modified, creating 4 measuring sites by drilling 2 holes to access the silver pads from outside. The modified cap was sterilized by means of UV rays for 24 h before each measuring session and stored under the sterile hood when not in use. Thus, the measuring cap was employed only during measurement sessions in order to avoid altering the sterility of the scaffold-sensor systems during culture in the incubator, for which the standard sterile cap of the multi-well was used. The overall setup is highlighted in Figure 1.

### 2.2. Experimental Design

Three-dimensional cell cultures—including scaffolds—were set up in 96-well sterile suspension culture U-bottom plates (Cellstar Greiner bio-one, Kremsmünster, Austria), in order to prevent the adhesion of hMSCs to the plastic substrate. In each plate only four wells in correspondence of the lid measurement sites were used. As hereinafter reported, in a preliminary study we established the optimal number of hMSCs to sow in each well (i.e., 200,000 cells/scaffolds). Therefore, we set up ten 96-well plates by assigning to each of the four available measuring sites a scaffold with its relative sensor, which remained associated to it until the end of the culture session. In order to determine the basal electrical signals of each system (i.e., scaffold/sensor) before seeding, we hydrated the scaffolds with complete culture medium (Iscove’s culture medium (σ) with 200 mM L-glutamine, penicillin 100 U/mL/streptomycin 100 μg/mL, 25 μg/mL fungizone, 0.6 UI/mL sodium heparin and 5% human platelet lysate) and then repositioned them back in the well including the sensor and in the presence of medium. Basal electrical signals were recorded after incubating the plates (5 h in a humidified incubator, 37 °C, 5% CO_2_). After the measurements, scaffolds and sensors were driedand exposed to air flow under the class A hood for 3 h and to UV rays for 12 h. Dry-seeding was thence realized the day after. Impedance measurements were performed on culture on days 3, 7, 10, 14, 17 and 21. Viability and cell proliferation were instead assessed on days 3, 7, 14 and 21 using a colorimetric enzymatic test. Control scaffolds for colorimetric testing and scaffolds for microscopy analysis were provided at all time points.

### 2.3. Impedance Measurements

Impedance measurements (magnitude and phase angle) were performed in a laminar flow hood (class A) to maintain sterility, using a commercial portable potentiostat (Palmsens PS Trace, PalmSens BV, Netherlands), with the protocol as previously optimized in [26,30]. A sinusoidal voltage with frequency linearly sweeping from 100 Hz to 10 kHz was applied to samples through the printed parallel carbon electrodes, and the current response was measured in that frequency range. In order to correlate changes in the measured impedance with the adhesion and growth of cells within the scaffold, we performed impedance measurements before and after cell seeding. All the pre-seeding tests were carried out using the very same type and quantity of medium that was used for cell culture, after leaving the scaffold in immersion for 1 h to allow the material to swell and to come in contact with the parallel electrodes, due to its volumetric expansion. The same measurements were repeated for all the sensors in order to obtain for each of them a reference value to be compared with the corresponding after-seeding measurement and also to assess the variability among the different scaffold/sensors systems.

Measurements after cell seeding were realized at specific time points (3, 7, 10, 14, 17 and 21 days), monitoring the variation of both impedance magnitude and phase angle during cell adhesion and growth with respect to frequency. Measured spectra were analyzed over all the range of frequencies considered, in order to identify the most suitable to enhance cell adhesion and growth monitoring. The same protocol was performed for a preliminary experiment with different cell numbers (50,000, 100,000, 200,000, 500,000 and 1,000,000 cells/scaffold) in order to assess the optimal amount, and then, the same measurements were repeated in parallel with biochemical viability assays using the optimized number of cells (i.e., 200,000 cells/scaffold).

### 2.4. Equivalent Circuit Modeling

Cell contribution to the overall acquired impedance spectra was extracted by fitting magnitude and phase angle spectra with an electronic equivalent circuit model; this approach was used to describe sensor-scaffold-cells system, adapting to the presented set up the models adopted in recent literature to model scaffold-based 3D cell cultures [34]. Parameters related to the contribution of scaffold and sensors were defined by modeling the pre-seeding acquisition performed in a range of frequencies from 100 Hz to 10 kHz considering only the culture medium and with culture medium and swelled cell-free scaffold. The parameters therefore included in the cell-free model were C_scaffold_, C_electrodes_, R_electrodes_, R_solution_, modeling respectively scaffold and electrodes electrical capacitances and electrodes and solution electrical resistances. After that, impedance spectra from cell-seeded scaffolds were modeled by integrating in the previous model the contribution of seeded hMSCs. The parameters added in the cell-seeded model were thus C_Cell,_ modelling cell membrane/intercellular capacitance, and R_Cell_, modelling cell resistance both in terms of cell membranes and intracellular cytoplasms. PS Palmsens Trace dedicated software was adopted for fitting the spectra and evaluating the variation of the extracted parameters at each time point.

### 2.5. hMSC Culture and Seeding

Bone-marrow-derived MSC from a single healthy donor were obtained, cultured, expanded and characterized, as previously described in [35]. All experiments were conducted with cells between passage 3 and 4. Cultures were trypsinized, washed and resuspended as single cells in a phosphate-buffered saline solution (PBS) with 5% of fetal bovine serum (5% FBS). Cell count and viability were performed in TRUCOUNT tubes (BD Biosciences, San Jose, CA, USA), by addition of 7-amino-actinomycin D (7-AAD) (BD Biosciences, San Jose, CA, USA), and analyzed using flowcytometry (FACS Canto Diva software byBD Bioscience, San Jose, CA, USA). After centrifugation, hMSCs were collected and resuspended at high cell density (5000 cells/μL) in complete culture medium (Iscove’s culture medium (σ) with 200 mM L-glutamine, penicillin 100 U/mL/streptomycin 100 μg/mL, 25 μg/mL fungizone, 0.6 UI/mL sodium heparin and 5% human platelet lysate). In the preliminary experiments, the cell concentration was adapted to have the required number of cells in a volume of 40 μL as needed. Dry scaffolds were placed in wells of 96-well sterile suspension culture F-bottom plates (Cellstar Greiner bio-one) for cell seeding. We adapted two static seeding methods for our experiment, namely “cold“ and “dry”, to increase the efficiency of cell penetration and distribution within the scaffold, by exploiting their characteristics; this approach was necessary since by using static seeding methods, most of the cells often adhered to the surface of the scaffold [36,37]. A small drop (40 μL—2 × 10^5^ viable hMSCs) was slowly deposited on top of dry cylindrical scaffold, waiting for the complete absorption of the drop. Any unabsorbed cell suspension was re-seeded on the scaffold. The plates were then covered and incubated for 1 h at 4 °C in the refrigerator and then for 2 h at 37 °C with 5% humidified CO_2_. The two incubations were performed without adding culture medium to improve cell adhesion. Every 30 min the plates were opened in sterile conditions, to re-seed any cell suspension present on the bottom of the well. The seeded scaffolds were then transferred with sterile forceps to the wells of the U-bottom culture plates previously set up with the corresponding sensors and conditioned in a humidified incubator (37 °C, 5% CO_2_). Only at this point, 150 μL of culture medium was added to each well with half fresh medium replacement every two days. Seeding wells were examined under an inverted microscope to check cell adhesion to the plastic.

On the days of the enzymatic test (days 3, 7, 14), the 96-well culture plates were replaced with new sterile plates to prevent the risk of contamination and get rid of any cellular debris adhered to the wells. In addition, the complete culture medium was completely replaced. To ensure correct humidity inside the plate, 12 peripheral wells were each filled with 200 μL of sterile water, restored if necessary.

### 2.6. hMSCs Enzymatic Proliferation Assay

To monitor hMSC proliferation during 3D culture, we used the Cell Counting Kit-8 colorimetric assay (CKK-8—Sigma-Aldrich, Merck Life Science S.r.l., 20149 Milano, Italy) based on the dehydrogenase activity detection in viable cells. Preliminary experiments with 2D culture in 96-well plates (2000 cells/cm^2^) were performed to test hMSCs’ response to CKK-8. In detail, 4-times treated hMSCs showed an average absorbance value of 15 ± 5% lower than cells at first-time treatment. Flow-cytometry counts confirmed a lower cell average (18 ± 5%) in cultures after 4-times treatments. Difference could be partially explained by the greater number of washes related to the 4 treatments, and however, it is in any case comparable to the variability range (16 ± 5%) observed between replicates with the same number of CKK-8 treatments (data not shown).

Moreover, we tested CKK-8 efficiency to detect a large number of cells in 3D culture, measuring the absorbance of 200,000 hMSCs/scaffold at the time of seeding (time 0) and after 14 and 21 days of culture (data not shown). Scaffolds were positioned in a new 96-well sterile suspension culture plate without sensors, covered with 200 μL of CKK-8 solution diluted (1 part CKK-8 + 10 parts of culture medium) and filtered (0.2 µm) to ensure sterility. The plates were then incubated (for 2 h 30′) in a humidified incubator (37 °C, 5% CO_2_). A cell-free control scaffold was incubated to determine the background measurement. 100 μL of solution was transferred into a 96-well F-bottom plate, and the absorbance at 450 nm was measured for each well using a microplate reader (ELX800, Bio Tek Instrument Inc, Winooski, VT 05404, USA). Cell proliferation was assessed on days 3, 7, 14 and 21 from sowing, and triple exposures of each sample were made under the same conditions. Before being put back into culture on the corresponding sensors, scaffolds were washed in new wells twice with 300 μL PBS 0.2% human albumin solution and once with 300 μL of culture medium (30′, humidified incubator 37 °C, 5% CO_2_).

Although CKK-8 product information reported “a very low cytotoxicity”, no standard indications were given about possible interferences during repeated exposures in the presence of 3D porous scaffold material. Therefore, the possible effect of the dye on cultures subjected to repeated CKK-8 treatments (up to 4) was also verified. We compared absorbance measurements in relation to the number of applications and culture days, to assess if any residues potentially trapped in the pores even after scaffold washes could exert any detrimental effects on cell viability and proliferation. For each measurement time point, we provided control scaffolds to be treated for the first time.

### 2.7. Immunostaining and Microscopy

This specific assessment required three different phases: fixation, permeabilization and blocking and imaging using fluorescence microscopy.

After removing culture medium, the scaffold was gently washed with PBS (Sigma-Aldrich, Merck Life Science S.r.l., 20149 Milano, Italy) and placed on a glass slide. The cylinder was then sectioned vertically in two parts and cut into thin slices (about 1 mm thick) starting from the side of the central cut. Each slice of scaffold positioned on a slide was then incubated (22 ± 2 °C, 10′) with 100 μL of 3.7% formaldehyde solution preheated to 37 ± 2 °C. Once the fixing solution was removed, the slices were washed with 100 μL of PBS and immediately removed.

Washed slices were permeabilized by adding 100 μL of 0.1% Triton X-100 solution (Sigma-Aldrich, Merck Life Science S.r.l., 20149 Milano, Italy) to 22 ± 2 °C for 5 min. Once the permeabilizing solution was removed, 2 rapid washes were carried out with 100 μL of PBS for each slice. The slices were subsequently incubated (22 ± 2 °C, 30′) with 3% FBS (fetal bovine serum—Sigma-Aldrich, Merck Life Science S.r.l., 20149 Milano, Italy) PBS solution.

Cell presence and distribution in the scaffold was visualized at 3, 7, 10, 14 and 21 days of culture by sacrificing the scaffolds and analyzing the thin slices with an inverted fluorescence microscope (Olympus IX70, OLYMPUS, 20034 Hamburg, Germany). The cell nuclei were stained with DAPI (4′,6-diamidino-2-phenylindole—Resnova). Thin slices were photographed at a magnification of 10×.

### 2.8. Statistical Analysis

To verify if the use of multiple KK-8 treatments influences hMSC proliferation, we used analysis of variance (ANOVA) techniques and the two-sample Student’s *t*-test. Whenever applicable, the Tukey HSD (honestly significant difference) procedure was used to test differences among two sample means, controlling the probability of making one or more type I errors. To investigate the trajectory of hMSC proliferation over time we fit a linear trend time series regression. To investigate the association between mean hMSC CKK-8 absorbance levels and mean impedance measurements at 3, 7, 14 and 21 days, we employed simple regression analysis techniques. The association between absorbance and impedance was further summarized using the Pearson’s correlation coefficient. We employed the Student’s t-test to evaluate the statistical significance of regression coefficients’ estimates. Statistical significance was set as *p* < 0.05 for the regression analyses.

## 3. Results and Discussion

### 3.1. hMSC Culture in 3D Hydrogel Scaffold

**Figure 2:**
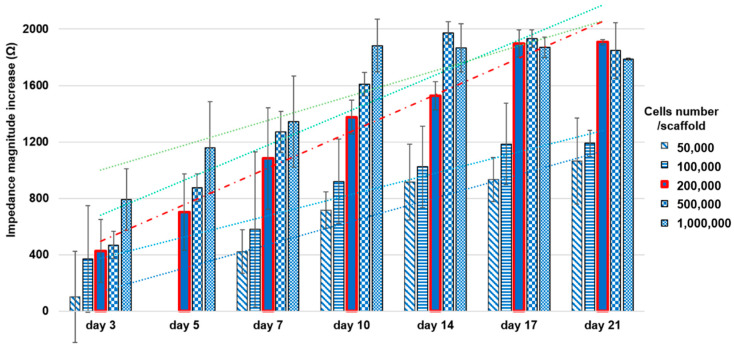
Preliminary impedance-based assessment for choosing the optimal cell concentration in the scaffold.

Results of the relationship between impedance measurements and number of cultured cells obtained from the preliminary pilot study comparing different cell concentrations (50,000, 100,000, 200,000, 500,000, 1,000,000) are summarized in Figure 2.

Regarding the impedance-based assay, results from the preliminary evaluation allowed 200,000 cells/scaffold to be selected as the optimal concentration to perform the comparison between this novel assay with the standardized cell proliferation enzymatic assay and the optical imaging. As highlighted in Figure 2, the higher concentrations (500,000 and 1,000,000 cells/scaffold), despite showing rapid growth during the first week, appeared to reach saturation after 14 days. This finding appeared to be in agreement with an acidification of the medium pH that could be observed during culture medium change after day 10, suggesting a condition of cell sufferance that is not ideal for a long-term healthy culture. On the other side, the lowest concentrations (50,000 and 100,000 cells/scaffold), despite showing a proliferation trend, reported a high variability indeed, probably due to the difficulty of seeding such a low cell number with high reproducibility in the different scaffolds. The condition with 200,000 cells/scaffold instead showed a better linearity (R^2^ = 0.9932), suggesting that this concentration is the best one to perform complete proliferation assays over a 21-day culture period.

### 3.2. Impedance-Based Cell Proliferation Monitoring

**Figure 3:**
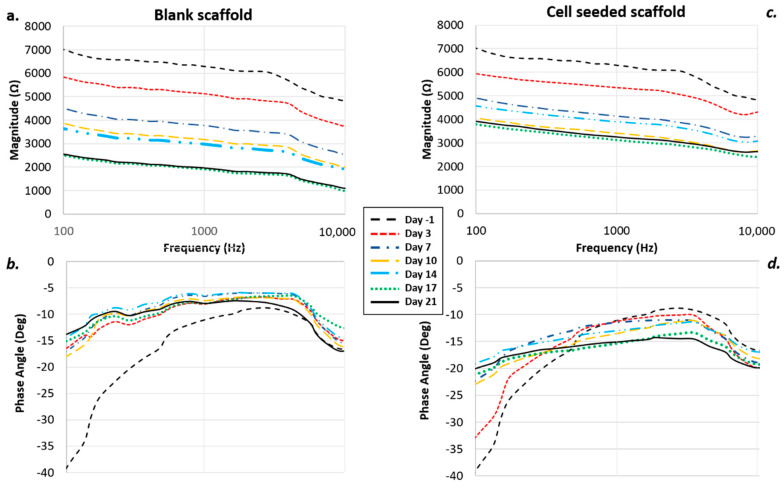
Impedance-based monitoring including controls (**a**) magnitude and (**b**) phase angle versus frequency) and seeded scaffolds (**c**) magnitude and (**d**) phase angle vs frequency.

Results from the preliminary experiments performed monitoring 200,000 cells/scaffold for 21 days using an impedance-based assay highlighted the possibility to adopt this approach to correlate a change in the electrical impedance parameters with the number of cells inside the scaffold. Comparing blank scaffolds to the hMSC-seeded ones, a decrease in the overall impedance can be highlighted in both conditions; this was probably due to the presence of the electrolytic medium, which was hydrating the hydrogel scaffold, enhancing the conductivity of the overall system. However, this conductivity increase appeared to be more enhanced in the blank scaffolds rather than in the cell seeded ones, due to the presence of the cells themselves that, spreading inside the pores of the scaffold, acted as insulating elements against the current flow within the culture medium (Figure 3).

**Figure 4:**
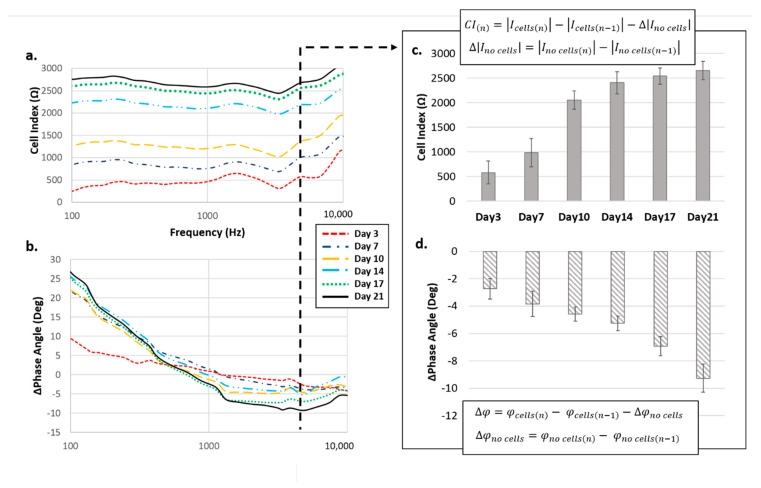
Cell index in terms of (**a**) magnitudeand (**b**) phase angle contribution to the overall impedance value. Results concerning 4 kHz frequency are specifically highlighted for both (**c**) magnitude and (**d**) phase angle.

The contribution of cells within the scaffold/electrolyte/cell system can be obtained by calculating the corresponding cell index (CI) in terms of both magnitude and phase angle, by subtracting the effect of the scaffold/electrolyte system at each specific time point, *n*, as detailed in the equation in Figure 4.

Due to the capacitive like behavior of cells—which act as insulators for lower frequencies and as conductors for higher frequencies—the CI was represented by an increase of magnitude, proportional to the amount of cells effectively attached to the scaffold, and by a decrease of the phase angle, particularly evident around 4 kHz. This specific value is perfectly in agreement with several works reported in the literature which highlighted this frequency as the optimal one for discriminating the contribution of cells with respect to the other elements that form the system [38].

### 3.3. Equivalent Circuit Modeling

**Figure 5:**
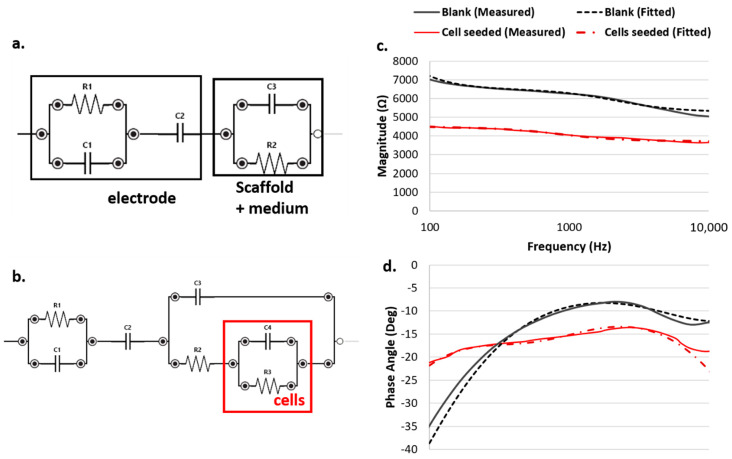
Equivalent circuit modeling the (**a**) blank and (**b**) cell seeded conditions: measured and fitted spectra for both the (**c**) module and (**d**) phase are reported.

The equivalent model adopted to fit the impedance spectra allowed the resistive and capacitive contribution of the cells to be highlighted, extracting the specific value of each parameter during the evolution of the cell culture. The best fitting was achieved with the equivalent model reported in Figure 5. By using this model, both the capacitive and the resistive behavior of the electrode and of the cells were enhanced.

Regarding the modeling circuit fitting the system without cells, we can highlight both a resistive and a capacitive behavior of the carbon-based electrode itself, as well as the typical double layer capacitance due to the interaction between the electrolyte/scaffold and the sensor itself. Additionally, most of the contribution in the impedance was given by the scaffold itself (i.e., a non-conductive material, so mainly expressing a capacitive behavior) and the medium (i.e., an electrolytic solution, which can be modeled as a resistor).

Comparing the conditions with and without cells, we observed that the presence of cells can be reliably modeled by adding parallel between a resistor and a capacitor (RC parallel) in series to the culture medium and in parallel to the scaffold. The cells attaching within the pores of the scaffold acted as series resistance with respect to the culture medium and in parallel to the scaffold material.

**Figure 6:**
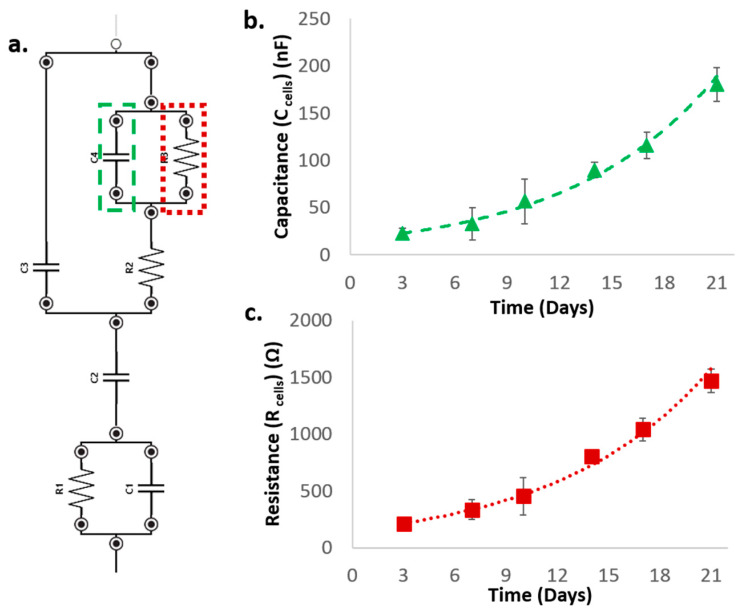
Evolution of the relevant parameters concerning the cell contribution during time (C_cells_, R_cells_). (**a**): Equivalent circuit with R_cells_ and C_cells_ highlighted, (**b**): Evolution of C_cells_ during culture, (**c**): Evolution of R_cells_ during culture.

As shown in Figure 6, an exponential increase of both R_cells_ and C_cells_ can be observed throughout the 21 days of culture, suggesting a proliferation of cells within the scaffold and thus an increase of both the resistive and the capacitive contribution to the overall impedance. This result appears to be in agreement with that reported by scientific literature [39]; in particular, the capacitance and resistance extracted from the equivalent circuit models are highlighted as useful parameters to describe cell proliferation in a 3D cell culture, since they closely relate to the overall cell number and volume.

### 3.4. Enzymatic Activity-Based Cell Proliferation Monitoring

Preliminary experiments performed to test the growth of different hMSC concentrations on hybrid gelatin-chitosan hydrogel scaffolds, indicated that the seeding concentration of 200,000 hMSCs/scaffold was the most suited to monitor cell proliferation in 21-day culture, both in terms of electrical impedance measurements and absorbance measurements.

**Figure 7:**
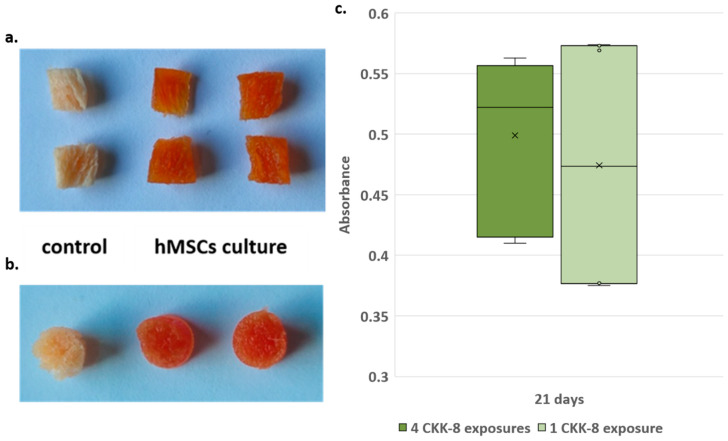
CKK-8 staining in 3D culture: (**a**) section and (**b**) upper-view of CKK-8 macroscopic staining of scaffolds, control (cell-less scaffold), hMSC culture (scaffold with cells after 14 days of culture); (**c**) box-plot of absorbance measurements at 21 days of culture. Distribution of absorbance measurements for scaffolds exposed to 4 CKK-8 treatments (x = average = 0.499 ± 0.062) and scaffolds exposed to 1 CKK-8 treatment (x = average = 0.474 ± 0.098), *p* = 0.64.

Macroscopic evaluation of CKK-8-stained scaffolds after 14 days of culture showed a uniform distribution of formazan dye, as evidenced by the vertical sections performed on the cylinders. This indicated that the CCK-8 solution had reached all parts of the scaffolds and that the cells were uniformly distributed along and inside the scaffold. Furthermore, the cell-free scaffold (control) shows that CKK-8 treatment did not cause any non-specific staining of the scaffold (Figure 7a,b). 

Results obtained by testing if repeated treatments (up to 4) over time with CKK-8 could affect cell viability showed no significant differences. This suggested that potentially trapped residues of the dye in the scaffold pores did not interfere with cell proliferation.

In detail, at 7 days of culture, absorbance measurements of scaffolds never treated with CKK-8 were compared with those that already had been at 3 days of culture. The statistical test did not reveal any differences between the two groups of scaffolds (*t*-test, *p* = 0.49). Similarly, the comparisons between scaffolds (1 vs. 2 vs. 3 CKK-8 treatments) at 14 days of culture did not show significant differences (ANOVA, *p* = 0.8; *t*-test 3 vs. 2, *p* = 0.78; *t*-test 3 vs. 1, *p* = 0.88). Similarly, 21-day culture comparisons, between 4-times treated scaffolds (3, 7, 14, 21 days) and the scaffolds that received less treatments did not show significant differences in the absorbance values (ANOVA, *p* = 0.54; *t*-test 4 vs less than 4, *p* = 0.84). Figure 7c shows the distribution of the absorbance measurements at 21 days of culture, for 4th CKK-8 exposure scaffolds and those at the first treatment. The statistical comparison, also in this case, showed no significant differences (*t*-test, *p* = 0.64); therefore, we considered all the scaffolds comparable regardless of the number of CKK-8 staining performed.

**Figure 8:**
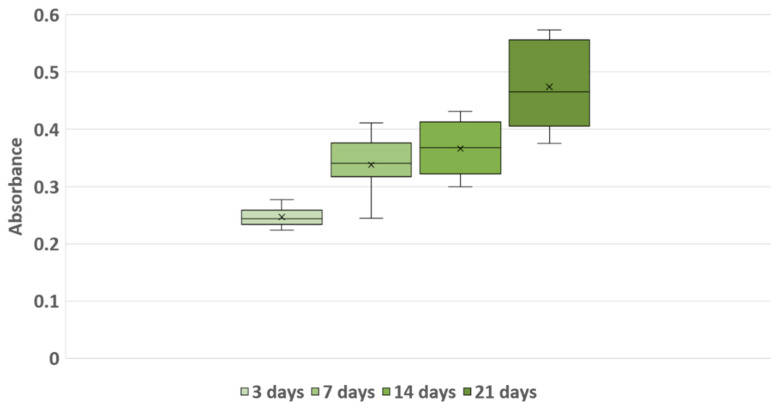
Box-plot of 3D culture proliferation detected with CKK-8 assay along the 21-day-long culture. X indicates samples average at each day.

Absorbance measurements obtained from CKK-8 at days 3, 7, 14, and 21 showed a progressive increase over time. In more detail, the mean absorbance values were 0.247 ± 0.015 (median value = 0.244), 0.338 ± 0.045 (median value = 0.341), 0.366 ± 0.044 (median value = 0.368) and 0.474 ± 0.075 (median value = 0.466) at 3, 7, 14 and 21 days of culture, respectively (Figure 8).

We performed a linear regression analysis of the absorbance measurements of the cultures over time to obtain the estimated daily growth rate in the first 21 days. The results suggested that the absorbance value increases by approximately 0.013 for every day in the scaffold (*p* < 0.001).

### 3.5. Association between Impedance Measurements and Biochemical Analysis

Results obtained from the regression analysis confirmed a valid relationship between the electrical impedance outcomes and the corresponding absorbance values of the proliferation assay. On the other hand, no significant relationship could be observed between the magnitude CI increase and the absorbance due to cell proliferation (*p* = 0.125). This result is expected since the estimation of the CI may be affected by additional variables related to cell adhesion and not directly linked to cell proliferation. Indeed, it is pointed out in [35] that CI is not directly related to the total cell number in the culture—like the absorbance value recorded from the enzymatic assay—but to newly attached cells, as they contribute to the overall magnitude because of the insulating composition of their membrane. In comparison, the results obtained by evaluating the relationship between phase angle measurements and the absorbance measurement indicated a significant negative association. More specifically, an increase of 0.01 in the cell proliferation resulted in a negative change of phase angle of −0.3 degrees (*p* = 0.019). The corresponding Pearson’s correlation coefficient was −0.981 (*p* = 0.019). These results can be supported by considering that phase angle decrease is positively associated with capacitance and negatively associated with resistance, and for these reasons, it is often taken as a key indicator for cell proliferation [40]. Thus, the higher phase angle suggests an increased quantity of intact cell membranes and an increase of the contribution of the cells to the overall impedance of the scaffold-cell system [41].

Considering the parameters modeling the contribution of the cells within the culture system, the results of the regression analysis indicated a significant association between both capacitance and resistance values with the extinction outcomes obtained with the enzymatic proliferation assay. More specifically, regarding the capacitance an increase of 0.01 in the cell proliferation resulted in a positive change of 6.5 nF (*p* = 0.044). The corresponding Pearson’s correlation coefficient was 0.956 (*p* = 0.044). Regarding the resistance, an increase of 0.01 in the cell proliferation resulted in a positive change of 51.6 Ω (*p* = 0.038). The corresponding Pearson’s correlation coefficient was 0.962 (*p* = 0.038). This relationship is well supported from related literature investigating 3D hMSC impedance-based monitoring with a very similar setup [39]. Thus, the capacitance and resistance extracted from equivalent circuit models appears to be closely related to the number/volume of live cells during proliferation, thus explaining the association with the extinction values obtained from the enzymatic proliferation assay.

### 3.6. Immunostaining and Microscopy

**Figure 9:**
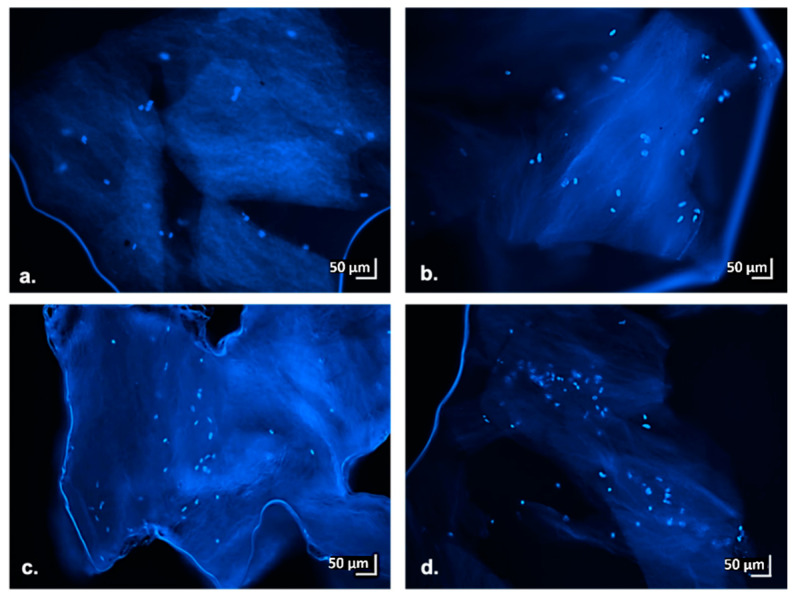
Fluorescent images of hMSCs on hybrid gelatin-chitosan hydrogel scaffolds at several days of culture. Thinly sliced portions of the central part of the scaffolds. Nuclei staining with DAPI (blue), 10× magnification. (**a**) 3 days of culture, (**b**) 7 days of culture, (**c**) 14 days of culture and (**d**) 21 days of culture.

In addition to the data obtained from CKK-8 colorimetric assay, the results achieved from the fluorescence microscopy analysis performed on scaffolds thin sections (at 3, 7, 14 and 21 day of culture) also confirmed the findings highlighted from the impedance measurements. In more detail, the images reported in Figure 9 show the presence of intact cell nuclei stained with DAPI; the number of identified nuclei specifically increased with increasing days of culture. These findings prove that scaffolds support hMSC growth over time. The microscopic data also confirms homogeneous cell distribution within the scaffold, as already highlighted by the sections macroscopic stained with CKK-8 (Figure 7a,b).

## 4. General Discussion and Future Developments

Overall, results obtained in terms of impedances appeared well supported by enzymatic assay outputs and by microscopy. Enzymatic tests, in fact, showed a continuous increase in average absorbance over the 21 days of culture, indicating that gelatin-chitosan hybrid hydrogel scaffolds well support hMSC cultures. Macroscopic staining and microscopic preparations of scaffolds confirmed a homogeneous distribution of cells within the volume of the scaffold starting from the first days of culture. Furthermore, the results of the regression analysis indicated a significant relationship between the extinction values of the enzymatic proliferation assay and respectively the phase angle (*p* = 0.019), C_cells_ (*p* = 0.044) and R_cells_ (*p* = 0.038). These findings support the possibility of associating the variation of those impedance-related parameters with the contribution of cells adhering onto the scaffold pores, thus affecting the overall impedance of the systems composed by seeded scaffold/medium/sensors. The results highlight the presented work as an interesting starting point for future investigation. As in any pilot study, there are—however—several limitations to be pointed out. Concerning the impedance-based measurements, a wide range of variability could be observed among the different independent systems evaluated (sensors/scaffold/culture medium). Most of them are due to the variability in samples geometry and the non-intrinsic conductivity of the adopted scaffold. Thus, the starting impedance of the non-seeded sensor-scaffold systems around 7 kΩ could represent a limiting factor for the overall sensitivity of the method. Only from a measuring perspective, the reproducibility and sensitivity could be improved through an enhanced standardization of the scaffold characteristics (e.g., porosity and dimensions) and of the conductivity of the overall system. On the other hand, results obtained from these scaffolds, non-conductive but already validated for regenerative medicine purposes in terms of cytocompatibility and porosity [10,11], are really promising and may lead to the direct adoption of this monitoring strategy for the optimization of 3D seeded scaffold ready for in vivo implantation.

Concerning the enzymatic reference method adopted, limitations are mainly related to the intrinsic additional complexities introduced while translating the reference methods from the 2D to the 3D environment, as highlighted in [42,43]. The commercial enzyme test used in this study was chosen for its low toxicity and ease of use, but obviously, it is not able to describe cell cultures in their complexity and in their interaction with the scaffold.

Considering the results and limitations, future developments will deal primarily with increasing the sensitivity and reducing the variability of the method presented. From a setup perspective, alternative designs and materials will be investigated to lower the overall impedance, to go beyond the monitoring of cell viability and proliferation, exploiting the potential of the presented method for also monitoring other events (e.g., differentiation or adhesion) which induce smaller signal variations. Gold standard methodologies, both invasive (e.g., DNA extraction, SEM analysis) or non-invasive (i.e., optical), will be adopted to confirm the possibility of associating those variations with cell morphological or phenotypical changes. From the signal processing perspective, additional strategies of denoising and feature extraction will be implemented to strengthen data analysis.

## 5. Conclusions

In conclusion, we presented here a non-invasive disposable modular system for monitoring cell proliferation in 3D scaffold-based cell cultures by means of impedance-based measurements. Flexible sensors were realized using AJP technology, depositing carbon-based ink on polyimide substrates specifically designed to fit into 96-well plates. After that, they were folded, sterilized and inserted in each well, together with the scaffold, to be monitored. Preliminary experiments confirmed the possibility to use this approach to monitor the proliferation of hMSCs seeded at different initial concentrations, highlighting the saturation of the growth curve, in agreement with optical and pH acidification of culture medium. Considering all this information and the maximum sensitivity achieved (200 Ω/day), 200,000 cells/scaffold was selected as the most suitable concentration for evaluating the repeatability of the methodology and to validate the output of the impedance-based assay with an enzymatic proliferation assay. Using this concentration, results obtained comparing impedances at 4 kHz throughout 21 days of culture showed a steady increase in the magnitude (total increase of 2.5 kΩ at day 21) and a decrease of the phase angle (total shift of −9 degrees at 21 days). The use of an equivalent circuit to model the scaffold-based culture allowed to highlight the specific contribution of cells in terms of capacitance and resistance at each time point to be extracted, highlighting an exponential increase of the resistance and capacitance during the 21 days of culture (with maximum values of 1.5 kΩ and 200 nF). Overall, the statistical association between outputs from the impedance-based method with the standard enzymatic assays supports the potential of the non-invasive approach proposed to perform a continuous monitoring of scaffold-based 3D cell cultures. Additionally, the intrinsic modularity and ease of customization of the approach proposed suggest the possibility to translate it in more complex 3D perfusion-based bioreactors. This would represent a promising scenario for obtaining useful feedback from environments mimicking physiological conditions, with interesting possible applications for regenerative medicine purposes, also from the perspective of optimizing the scaffold-culture system.
